# Sevoflurane is associated with reduced EGFR-VEGF-AKT signaling in glioma tissues relative to propofol: a bioinformatics and clinical pilot study

**DOI:** 10.3389/fonc.2026.1897789

**Published:** 2026-08-18

**Authors:** Qian Hu, Feng Lu, Jia-Sheng Wang, Rui-Yang Cao, Wen-Kai Liu, Ming-Ling Guo, Yong Lin, Mao-Lin Zhong, Wei-Dong Liang, Li-feng Wang

**Affiliations:** 1 The First Clinical Medical College of Gannan Medical University, Ganzhou, Jiangxi, China; 2 Department of Anesthesiology, The First Affiliated Hospital of Gannan Medical University, Ganzhou, Jiangxi, China; 3 Ganzhou Key Laboratory of Anesthesiology, The First Affiliated Hospital of Gannan Medical University, Ganzhou, Jiangxi, China

**Keywords:** angiogenesis, EGFR/VEGF/AKT pathway, gene expression, glioma, sevoflurane

## Abstract

**Purpose:**

This study aims to investigate the effects of sevoflurane on glioma-associated angiogenesis and to explore the potential mechanisms, providing preliminary experimental evidence for its possible association with molecular changes relevant to glioma angiogenesis.

**Methods:**

The GSE179004 dataset was downloaded from the GEO database. This dataset consists of three glioma samples treated with sevoflurane and three untreated control samples. Differential expression analysis was performed using the limma package (|logFC|>1, *P* < 0.05). Candidate angiogenesis-related differentially expressed genes (ARDEGs) were identified by intersecting DEGs with angiogenesis-related genes from GeneCards, CTD, and OMIM. GO and KEGG enrichment analyses were conducted. After Benjamini-Hochberg correction, none of the 36 angiogenesis-related candidate genes reached statistical significance, reflecting the limited statistical power of the small discovery dataset. These findings should be interpreted as hypothesis-generating rather than confirmatory. A PPI network was constructed using STRING, and hub genes were identified using CytoHubba in Cytoscape. A ceRNA network was also established. Hub gene expression was validated using GEO and TCGA datasets. In the clinical study, patients received sevoflurane inhalation anesthesia throughout surgery. Human glioma tissue samples were collected intraoperatively, and expression of hub genes and VEGF-AKT pathway components was verified by qRT-PCR and Western blot.

**Results:**

A total of 1,245 DEGs were identified (918 upregulated, 327 downregulated), from which 36 candidate ARDEGs were screened. Enrichment analysis showed these ARDEGs were significantly enriched in smooth muscle cell proliferation and the PI3K-AKT pathway (adj.*P* < 0.05, FDR<0.25). Seven hub genes were identified: *EGFR*, *AGT*, *EGR1*, *HBEGF*, *ITGA2*, *FGFR2*, and *NPY*. Validation in the GSE179004 dataset showed that *ITGA2* and *FGFR2* were significantly upregulated, while *EGR1* and *HBEGF* were downregulated by sevoflurane (*P* < 0.05). TCGA analysis revealed that *EGFR* expression was significantly higher in glioma tissues than in normal tissues (*P* < 0.001) and was associated with poor prognosis (HR = 1.364, 95% CI: 1.074-1.734, *P* = 0.011). qRT-PCR on clinical samples demonstrated that, compared to the propofol group, the sevoflurane group had significantly increased expression of *ITGA2*, *NPY*, and *FGFR2*, and significantly decreased expression of *EGFR*, *AGT*, *EGR1*, *HBEGF*, and *VEGFA* (*P* < 0.05). Western blot confirmed that protein levels of EGFR, VEGF, AKT, and p-AKT were significantly reduced in the sevoflurane group (*P* < 0.05). No significant differences in baseline characteristics or intraoperative parameters were observed between the two groups (*P >*0.05).

**Conclusion:**

This exploratory pilot study compared sevoflurane inhalational anesthesia and propofol intravenous anesthesia in human glioma tissues. Sevoflurane reduced EGFR, VEGFA and p-AKT expression, indicating distinct regulation of the EGFR-VEGF-AKT pathway versus propofol. Notably, both agents have inherent anti-tumor and anti-angiogenic effects, so these expression changes reflect relative intergroup differences rather than absolute declines from untreated baseline. Limited by small sample size, no long-term follow-up, missing functional angiogenesis tests and no anesthesia-free controls, these findings are only hypothesis-generating and need validation in larger prospective trials with proper controls.

**Clinical trial registration:**

www.chictr.org.cn, identifier ChiCTR2400092575

## 1 Introduction

Gliomas are the most common primary tumors of the central nervous system, accounting for 70–80% of all malignant brain tumors. Globally, approximately 6 out of every 100,000 people are diagnosed with glioma each year (1–3). According to the World Health Organization (WHO) classification of central nervous system tumors, gliomas are categorized into four grades (I–IV) (4). WHO grade I gliomas are well-differentiated, slow-growing, and weakly invasive; they are largely benign, and patients generally have an excellent prognosis after complete surgical resection. WHO grade II gliomas grow infiltratively, have a prolonged clinical course, and are prone to recurrence after surgery with a potential risk of progression to higher-grade lesions. WHO grade III anaplastic gliomas exhibit significantly higher malignancy, marked cellular atypia, active mitotic activity, strong invasiveness, and rapid disease progression; surgery alone offers limited therapeutic benefit. WHO grade IV glioblastoma is the most malignant and highly invasive, associated with an extremely poor prognosis. Among these, glioblastoma (GBM) is the most malignant and prognostically poor tumor of the central nervous system, with a median survival of only 14.6–16 months (5). Over the past several decades, no significant advances have been made in the treatment of GBM. The current standard of care for GBM involves maximal safe surgical resection, followed by radiotherapy within 30 days post-surgery and chemotherapy with temozolomide (6). However, these therapeutic approaches often fail to completely eradicate the tumor, leading to recurrence and poor prognosis, with a 5-year survival rate of only approximately 10% (7).

Angiogenesis, the process of new blood vessel formation, is a complex and dynamic process regulated by various pro-angiogenic and anti-angiogenic molecules. It plays a crucial role in tumor growth, invasion, and metastasis (8). Angiogenesis is essential for tumor progression and metastasis, serving not only as a core channel for nutrient and oxygen supply but also as a mediator of signals such as cytokines and growth factors that directly promote tumor enlargement and invasive spread (9). Gliomas are characterized by a high degree of vascularization. Studies have shown that their vascular density is significantly higher than that of other types of brain tumors, facilitating rapid tumor expansion and invasion into surrounding healthy tissue (10). Vascular endothelial growth factor (VEGF) and its mediated signaling pathways occupy a central position in the complex regulatory network of glioma angiogenesis. As a key regulator of angiogenesis, VEGF binds to receptors on the surface of endothelial cells, activating downstream signaling pathways such as PI3K/Akt and Ras/MAPK, which in turn regulate endothelial cell proliferation, migration, and new blood vessel formation (11).

Angiogenesis is driven by multiple signaling pathways. The EGFR-VEGF-AKT axis, in particular, plays a critical role in gliomas. Activation of the EGFR upregulates VEGF expression, which in turn stimulates AKT signaling to promote endothelial cell survival, proliferation, and migration. This cascade is frequently hyperactivated in gliomas due to EGFR gene amplification, overexpression, or activating mutations, and it is associated with higher tumor grade and poor prognosis.

Anti-angiogenic therapy represents a significant avenue of exploration in the field of targeted therapy for glioma. Bevacizumab, the first anti-vascular endothelial growth factor agent to enter clinical application, has shown promising potential in the treatment of glioma (12). It exerts its effects by binding to VEGF and blocking its downstream signaling, thereby inhibiting angiogenesis. Clinical studies have demonstrated that bevacizumab, when used alone or in combination with radiotherapy and chemotherapy in glioma patients, particularly those with recurrent glioma, could significantly prolong progression-free survival (PFS). However, its benefit in improving overall survival (OS) remains limited (13, 14). Furthermore, long-term use of bevacizumab often induces therapeutic resistance, with approximately 30%–60% of patients experiencing tumor recurrence after 6–12 months of continuous treatment (15). This resistance may be attributed to the complexity and adaptive remodeling of the tumor microenvironment, through which tumors evade immune surveillance and drug action via multiple compensatory mechanisms (16). In addition, targeting other angiogenesis-related signaling pathways including those mediated by basic fibroblast growth factor (bFGF), platelet-derived growth factor (PDGF), and hypoxia-inducible gene 2 (HIG2) has also demonstrated considerable potential (17). Meanwhile, alongside advances in genomics and proteomics, personalized treatment strategies based on individual molecular profiles are under active investigation (18). In summary, while anti-angiogenic therapy has yielded preliminary advancements in glioma treatment, substantial challenges persist, such as inadequate efficacy and a high rate of drug resistance. These barriers highlight the pressing need to further investigate the mechanisms underlying glioma angiogenesis and develop innovative therapeutic approaches.

Sevoflurane is a commonly used inhalational anesthetic that not only has widespread applications in anesthesiology but has also demonstrated anti-tumor properties in various cancers (19). Recent studies indicate that sevoflurane affects tumor angiogenesis through multiple mechanisms. For instance, it downregulates the expression of vascular endothelial growth factor (VEGF) in breast cancer mouse models, reducing both VEGF protein levels and mRNA expression, thereby inhibiting angiogenesis (20, 21). Similarly, sevoflurane has been shown to suppress hypoxia-induced lung cancer cell growth and metastasis by modulating the hypoxia-inducible factor-1α (HIF-1α) signaling pathway, leading to a significant decrease in the expression of its downstream target gene VEGF (22).

In this study, we adopted a multi-stage translational approach that integrated bioinformatic screening, *in vitro* validation, and clinical tissue validation. Bioinformatics analysis and screening tools were used to generate a set of candidate genes. To validate the hub genes and key proteins in the EGFR-VEGF-AKT pathway identified through this approach, we performed qRT-PCR and Western blot analyses on human glioma tissue samples. We assessed the mRNA levels of seven hub genes (*EGFR*, *AGT*, *EGR1*, *HBEGF*, *ITGA2*, *NPY*, *FGFR2*) and *VEGFA*, as well as the protein levels of EGFR, VEGFA, AKT, and p-AKT. These experimental validations were intended to assess the biological relevance of the prioritized candidates in a clinically relevant context. Within this framework, we aimed to investigate the effect of sevoflurane on glioma-associated angiogenesis and to explore its underlying molecular mechanisms.

## 2 Materials and methods

### 2.1 Data download and searching the OMIM

The GSE179004 dataset (23) was downloaded from the GEO database (https://www.ncbi.nlm.nih.gov/gds) using the R package GEOquery. This dataset, derived from postoperative human glioblastom tissues, was generated on the GPL23126 platform, with detailed information provided in **Table 1**. The GSE179004 dataset contains three sevoflurane-treated glioblastom samples (Sevoflurane group) and three control samples (Control group). The control group consisted of glioblastom tissue samples from patients who did not receive sevoflurane (anesthesia with other agents, details not specified in the original GEO submission).

**Table 1:** Summary of the GEO microarray dataset.

Dataset	GSE179004
Platform	GPL23126
Species	*Homo sapiens*
Tissue	Glioma
Sevoflurane-treated samples	3
Control samples	3
Reference	PMID: 36856519

Angiogenesis-related genes (ARGs) were collected from the GeneCards database (24) (https://www.genecards.org/). The GeneCards database provides comprehensive information on human genes. Using “Angiogenesis” as the search keyword and retaining only protein-coding genes with a Relevance Score > 2, a total of 701 ARGs were obtained. Additionally, using the same keyword “Angiogenesis” in the CTD database (25) (http://ctdbase.org/) yielded 40 ARGs, and searching the OMIM database (26) (http://omim.org/) identified 189 ARGs. After merging and removing duplicates, a total of 785 ARGs were compiled for subsequent analysis.

The GSE179004 dataset was preprocessed using the R package limma (27), which included standardization, probe annotation, and normalization.The limma settings were as follows: normalization, moderation, and Benjamini-Hochberg adjustment for multiple testing. Principal Component Analysis (PCA) (28) was subsequently performed on the resulting expression matrix.

### 2.2 Identification of sevoflurane-associated ARDEGs in gliomas

Differential expression analysis between sevoflurane-treated glioma samples and controls was performed using the R package limma. In this discovery-oriented screening step, genes with |logFC| > 1 and *P* < 0.05 (nominal) were initially identified as candidate differentially expressed genes (DEGs). This liberal threshold was deliberately chosen to maximize the capture of potential gene expression signals following sevoflurane treatment. Genes with |logFC| > 1 and *P* < 0.05 were additionally highlighted as DEGs with larger effect sizes. It should be emphasized that this lenient filtering criterion was intended to serve a “candidate pool” function, facilitating the subsequent functional focusing step, rather than to provide a definitive list of robustly validated DEGs. The Benjamini-Hochberg procedure was applied for *P*-value adjustment. The results of the differential expression analysis were visualized using a volcano plot generated with the R package ggplot2.

Angiogenesis-related differentially expressed genes (ARDEGs) were identified by intersecting the candidate DEGs with the previously compiled list of 785 ARGs. This intersection effectively filtered out approximately 97% of the initial candidate DEGs, retaining only 36 ARDEGs for subsequent functional enrichment and network analyses. The biological relevance of these prioritized candidates was then assessed through multi-layered validation, including PPI network analysis, independent TCGA cohort validation, and experimental verification in clinical samples. A volcano plot was generated using the R package ggplot2 to visualize the DEGs, with the subsequently identified hub genes highlighted. Additionally, a heatmap of the ARDEGs was created using the R package pheatmap.

### 2.3 GO and KEGG pathway enrichment analysis

Gene Ontology (GO) analysis (29) is a commonly used method for large-scale functional enrichment studies, encompassing three categories: biological process (BP), cellular component (CC), and molecular function (MF). The Kyoto Encyclopedia of Genes and Genomes (KEGG) (30) is a widely used database that integrates information on genomes, biological pathways, diseases, and drugs. GO and KEGG enrichment analyses of the ARDEGs were performed using the R package clusterProfiler (31). Statistically significant terms were identified based on the criteria of adj. *P* < 0.05 and a false discovery rate (FDR, q value) < 0.25, with *P*-values adjusted using the Benjamini-Hochberg method.

### 2.4 PPI network construction and hub gene screening

A protein-protein interaction (PPI) network illustrates the physical interactions between proteins, which play crucial roles in various biological processes, including signal transduction, gene expression regulation, energy and substance metabolism, and cell cycle control. Systematic analysis of these interactions is essential for understanding protein function within biological systems, elucidating the mechanisms underlying physiological and pathological states (such as disease), and exploring functional relationships between proteins. The STRING database (32)(https://string-db.org/)is a comprehensive resource for searching known and predicted protein-protein interactions. In this study, a PPI network for the ARDEGs was constructed using the STRING database, with a minimum required interaction score of > 0.400. Highly interconnected clusters within a PPI network may represent molecular complexes with specific biological functions. Therefore, genes exhibiting interactions with other genes within the network were selected for subsequent analysis. To identify hub genes, five algorithms from the CytoHubba plugin in Cytoscape were applied: Maximal Clique Centrality (MCC) (33), Degree, Stress, Edge Percolated Component (EPC), and Closeness. For each algorithm, the ARDEGs were scored, and the top 10 genes were selected based on their rankings. The results from the five algorithms were then intersected, and the overlapping genes were visualized using a Venn diagram. These intersecting genes were ultimately identified as the hub genes.

### 2.5 Construction of the ceRNA regulatory network

The miRcode database iRcode (34) was used to identify potential interactions between long non-coding RNAs (lncRNAs) and microRNAs (miRNAs). Subsequently, the StarBase database (35) was employed to predict miRNA-mRNA interactions. Based on these data, a competing endogenous RNA (ceRNA) regulatory network, consisting of lncRNA-miRNA-mRNA interactions, was constructed. The resulting network was then visualized using Cytoscape software.

### 2.6 Validation of hub gene expression

To further investigate the differential expression of the hub genes between sevoflurane-treated glioma samples and control samples in the GSE179004 dataset, group comparison plots were generated based on the expression levels of these hub genes. Additionally, glioma gene expression data and corresponding clinical information were downloaded from The Cancer Genome Atlas (TCGA) database (36)(https://portal.gdc.cancer.gov/). These data were used to analyze the expression levels of the hub genes in glioma tissues compared to normal tissues, as well as to evaluate the association between hub gene expression and the survival outcomes of glioma patients.

### 2.7 Single-gene analysis of EGFR

The expression levels of *EGFR* were compared between glioma tissues and normal tissues. Subsequently, receiver operating characteristic (ROC) curve analysis was performed to assess the diagnostic performance of EGFR in distinguishing glioma from normal tissues, and the area under the curve (AUC) was calculated. Furthermore, logistic regression analysis was conducted to assess the association between *EGFR* expression levels and various clinicopathological features in glioma patients, including sex, age, disease-specific survival, progression-free interval events, isocitrate dehydrogenase (IDH) mutation status, WHO grade, and glioma subtype.

### 2.8 Collection of glioma tissue samples

#### 2.8.1 Study design

This study was approved by the Ethics Committee of the First Affiliated Hospital of Gannan Medical University (Approval No. LLSC-2024-211) and registered with the Chinese Clinical Trial Registry on November 19, 2024 (ChiCTR2400092575). The study was conducted in accordance with the principles of the Declaration of Helsinki. Written informed consent was obtained from all patients or their legal guardians.

#### 2.8.2 Study participants

A total of 20 adult patients (male and female) who were scheduled to undergo elective craniotomy under general anesthesia and were diagnosed with glioma by magnetic resonance imaging (MRI) were enrolled in this study. The patients were divided into two groups based on the anesthetic regimen: the propofol group (Group P), which received propofol combined with remifentanil, and the sevoflurane group (Group S), which received sevoflurane combined with remifentanil.

#### 2.8.3 Inclusion criteria

Patients were enrolled in the study if they met all the following inclusion criteria:

1. Scheduled to undergo elective intracranial resection of glioma at our hospital, aged between 18 and 80 years.
2. Classified as American Society of Anesthesiologists (ASA) physical status I–III.
3. Glasgow Coma Scale (GCS) score of 15, with no symptomatic elevation of intracranial pressure preoperatively.
4. Body mass index (BMI) between 18.5 and 28 kg/m^2^.
5. Provided written informed consent after being fully informed about the study.

#### 2.8.4 Exclusion criteria

Patients were excluded from the study if they met any of the following criteria:

1. Diagnosed with traumatic brain injury, intracerebral hemorrhage, cerebral infarction, or other cerebrovascular diseases.
2. History of alcohol or drug abuse, or a personal or family history of psychiatric disorders.
3. Uncontrolled hypertension or severe cardiac disease impairing cardiac function (New York Heart Association [NYHA] functional class ≥ III).
4. Known allergy to anesthetic agents.
5. Pregnancy or breastfeeding.
6. Received radiotherapy or chemotherapy prior to surgery.
7. Participation in other clinical trials, refusal to participate in this study, or any other condition deemed unsuitable for participation by the investigators.

#### 2.8.5 Withdrawal criteria

1. Unforeseeable complications occurred during surgery, including but not limited to major hemorrhage, embolic events, or injury to other organs.
2. The patient requested to withdraw from the study during the trial period.

#### 2.8.6 Randomization and blinding

Eligible subjects were randomized using a block randomization method in SPSS. A random number seed of 20241119 was set, and a random sequence was generated using the software’s built-in generator. The block length was 4, with a total of 5 blocks. Within each block, subjects were ranked by the generated values; the first two were allocated to the sevoflurane group (Group S), and the last two to the propofol group (Group P). The randomization results were sealed in sequentially numbered, opaque envelopes by a researcher not involved in the study. The allocation scheme was also prepared as randomization cards by another independent researcher, placed in sequentially numbered, opaque envelopes to ensure allocation concealment.

After the patient entered the operating room, the anesthesia provider opened the envelope and performed the assigned protocol. The anesthesiologist responsible for drug administration took no part in other trial procedures and only managed intraoperative medications. Another blinded anesthesiologist recorded all intraoperative data. All surgeries were performed by the same surgeon. Collected glioma tissue samples were coded, and a researcher not involved in the clinical grouping or laboratory testing used a computer-generated random sequence to independently select five cases with postoperative pathological confirmation of glioblastoma (WHO grade IV) from each of the propofol and sevoflurane groups for subsequent qRT-PCR and Western blot analyses. The remaining samples were banked for future studies. The personnel performing laboratory tests (qRT-PCR, Western blot) as well as those responsible for data entry and statistical analysis were blinded to group allocation.

In the event of an emergency, the treating physician or researcher could unblind the group assignment for the relevant patient to ensure patient safety, and the patient would subsequently be withdrawn from the study.

#### 2.8.7 Anesthesia protocol

##### 2.8.7.1 Anesthesia induction

All patients fasted for 6 hours and abstained from clear liquids for 2 hours prior to surgery, receiving no premedication. Anesthesia was administered by the same anesthesiologist for all patients. Upon arrival in the operating room, an intravenous line was established, and patients were connected to monitors for bispectral index (BIS), automated non-invasive blood pressure (NIBP), electrocardiogram (ECG), heart rate (HR), pulse oxygen saturation (SpO_2_), respiratory rate (RR), and body temperature. Under local anesthesia, radial artery catheterization was performed for invasive mean arterial pressure (MAP) monitoring and blood gas analysis. General anesthesia was induced with tracheal intubation. Based on ideal body weight, the induction agents were administered sequentially as follows: midazolam 0.05 mg/kg, sufentanil 0.5 μg/kg, propofol 2 mg/kg, and rocuronium 0.6 mg/kg. After adequate muscle relaxation was achieved, tracheal intubation was performed under video laryngoscope guidance. Following intubation, mechanical ventilation was initiated with the following parameters: tidal volume 6–8 mL/kg, respiratory rate 12–16 breaths/min, inspiratory-to-expiratory ratio 1:2, and oxygen flow rate 2 L/min, to maintain end-tidal carbon dioxide pressure (PetCO_2_) between 30 and 45 mmHg. Subsequently, right internal jugular vein catheterization was performed under ultrasound guidance. Additionally, scalp nerve blocks were performed using 0.375% ropivacaine (2 mL each for the bilateral supraorbital, supratrochlear, auriculotemporal, and zygomaticotemporal nerves; 5 mL each for the greater and lesser occipital nerves).

##### 2.8.7.2 Anesthesia maintenance

Following induction, anesthesia was maintained according to the preoperative group allocation: Group P: Propofol at 6–8 mg/kg/h and remifentanil at 0.05-0.2 μg/kg/min. Group S: Sevoflurane at 1 minimum alveolar concentration (MAC; 1.71%) and remifentanil at 0.05-0.2 μg/kg/min. Based on hemodynamic parameters, the anesthesiologist administered supplemental sufentanil (5-10 μg) during pinion head-holder application or skin incision to attenuate intense stimuli. The doses of anesthetics were adjusted to maintain MAP and HR within ± 20% of baseline values and to keep the BIS value between 40 and 60. Intermittent boluses of rocuronium were administered as needed to maintain muscle relaxation. All anesthetics were discontinued at the end of surgery. Postoperatively, all patients received patient-controlled intravenous analgesia (PCIA). The PCIA solution was prepared as follows: sufentanil 2 μg/kg and ondansetron 8 mg, diluted with 0.9% sodium chloride to a total volume of 100 mL.

##### 2.8.7.3 Intraoperative adverse events and rescue measures

Hemodynamic stability was maintained during surgery. When MAP decreased by more than 20% from baseline, norepinephrine was administered, and the infusion rate was adjusted according to the blood pressure response. When MAP increased by more than 20% above baseline, after excluding inadequate analgesia or inappropriate depth of anesthesia, antihypertensive treatment with nitroglycerin or nimodipine was applied. When HR fell below 50 beats/min, atropine 0.5 mg was given intravenously; when HR exceeded 100 beats/min, after excluding relevant triggers, esmolol was administered. Intraoperative fluid replacement was adjusted in real time based on blood loss, urine output, and pulse pressure variation (PPV).

##### 2.8.7.4 Outcome measures and variables

Patient baseline characteristics and intraoperative parameters were monitored, including sex, age, ECG, NIBP, HR, SpO_2_, RR, PetCO_2_, BIS, fluid infusion volume, blood loss, and urine output. The following variables were recorded at specific time points: upon arrival in the operating room (T_0_), before the start of surgery (T_1_), at the start of surgery (T_2_), at the time of dural incision (T_3_), at the time of glioma resection (T_4_), at the start of cranial closure (T_5_), and at the end of surgery (T_6_). The recorded variables included MAP, HR, intraoperative use of vasoactive agents, and remifentanil consumption. Glioma tissue samples were collected within 30 minutes of resection, immediately frozen in liquid nitrogen, and subsequently stored at -80 °C for further analysis. The prespecified exploratory laboratory outcomes included: relative mRNA expression levels of the seven hub genes (*EGFR, AGT, EGR1, HBEGF, ITGA2, NPY, FGFR2*) and *VEGFA*, measured by qRT-PCR; and relative protein expression levels of EGFR, VEGFA, AKT, and p-AKT, measured by Western blot.

### 2.9 Quantitative real-time PCR (qRT-PCR)

Total RNA was isolated from tissue samples using TRIzol reagent and subsequently purified with an RNA ultra-pure extraction kit. RNA concentration and purity were assessed using a NanoDrop 2000 spectrophotometer (Thermo Fisher) by measuring A260/A280 and A260/A230 ratios. Only samples with A260/A280 between 1.8 and 2.0 and A260/A230 > 2.0 were used. Purified RNA was then reverse transcribed into complementary DNA (cDNA) using the ReverTra Ace qPCR RT Kit. Amplification and detection were performed using a real-time quantitative PCR system under the following cycling conditions: pre-denaturation at 94 °C for 30 s, followed by 40 cycles of denaturation at 95 °C for 5 s, annealing at 58 °C for 15 s, and extension at 72 °C for 10 s. In this study, β-actin was used as the internal reference gene. The relative expression levels of the target genes were calculated using the **2^^(-ΔΔCt)^** method, with β‑actin as the internal reference gene. The primer sequences for all target genes are listed in **Table 2**. Given the exploratory nature of the molecular analyses and the testing of multiple mRNA and protein targets, we applied the Holm-Bonferroni correction for multiple comparisons to control the family-wise error rate. Both raw and Holm-adjusted *P*-values are reported for all molecular outcomes. Effect sizes (mean differences) and 95% confidence intervals are also provided. Given the small sample size (n = 5 per group), these results should be interpreted as exploratory.

**Table 2:** Primer sequences for qRT-PCR.

Gene	Primer Sequence (5’→3’)
β-Actin-F	5’-AGCATCCCCCAAAGTTCACAA-3’
β-Actin-R	5’-TGGGGTGGCTTTTAGGATGG-3’
AGT-F	5’-GCAGATAACAACCCCGGACA-3’
AGT-R	5’-AGACCAAGGAGAAACGGCTG-3’
EGFR-F	5’-GCCTTGACTGAGGACAGCAT-3’
EGFR-R	5’-CCTTACGCCCTTCACTGTGT-3’
EGR1-F	5’-TCCCATTTACTCAGCGGCAC-3’
EGR1-R	5’-TGGAAACAGGTAGTCGGGGA-3’
FGFR2-F	5’-TCCGAGAATACCTCCGAGCC-3’
FGFR2-R	5’-TCTCTGGCGAGTCCAAAGTC-3’
HBEGF-F	5’-CATCGTGGGGCTTCTCATGT-3’
HBEGF-R	5’-ACTGGGGACGAAGGAGTCTT-3’
ITGA2-F	5’-TTCCACACCCCATCTTGCTC-3’
ITGA2-R	5’-ATCGCCCCCTCTCCTAACTT-3’
NPY-F	5’-CAACCTCATCACCAGGCAGA-3’
NPY-R	5’-CTTCAAGCCGAGTTCTGGGA-3’
VEGFA-F	5’-ACCAAGGCCAGCACATAGG-3’
VEGFA-R	5’-ATTAGACAGCAGCGGGCAC-3’

### 2.10 Western blot analysis

Tissue samples from each group were lysed in lysis buffer (RIPA buffer: 50 mM Tris-HCl, pH 7.4, 150 mM NaCl, 1% NP-40, 0.5% sodium deoxycholate, 0.1% SDS) supplemented with 1 mM PMSF (protease inhibitor) and PhosSTOP phosphatase inhibitor cocktail (Roche) and thoroughly homogenized using a grinder at 65 Hz for 60 s. The homogenate was centrifuged at 12,000 rpm for 10 min at 4 °C. The supernatant was carefully collected and transferred to a new EP tube for protein concentration determination, while the pellet was discarded. Protein concentration in the supernatant was measured using a BCA assay kit, and a standard curve was generated. The supernatant was then mixed with loading buffer and boiled for 5 min to obtain total protein, which was subsequently stored at -20 °C. Protein samples were separated by SDS-PAGE and transferred onto a PVDF membrane. The membrane was blocked with 3% non-fat milk for 1 h at room temperature. The membrane was then incubated with primary antibodies for 2 h at room temperature, followed by incubation with horseradish peroxidase (HRP)-conjugated secondary antibodies for 2 h at room temperature. After each incubation, the membrane was washed three times with washing buffer. The following primary and secondary antibodies were used: Internal control: Rabbit Anti-β-Actin (81115-1-RR, Proteintech, 1:2000); secondary antibody: HRP-Goat Anti-Rabbit IgG (H + L) (RGAR001, Proteintech, 1:2000). Target proteins: Rabbit Anti-EGFR (18986-1-AP, Proteintech, 1:2000); secondary antibody: HRP-Goat Anti-Rabbit IgG (H+L) (RGAR001, Proteintech, 1:2000); Rabbit Anti-VEGFA (10745-1-AP, Proteintech, 1:5000); secondary antibody: HRP-Goat Anti-Rabbit IgG (H + L) (RGAR001, Proteintech, 1:2000); Rabbit Anti-AKT (10176-2-AP, Proteintech, 1:2000); secondary antibody: HRP-Goat Anti-Rabbit IgG (H + L) (RGAR001, Proteintech, 1:2000); Mouse Anti-p-AKT (66444-1-Ig, Proteintech, 1:2000); secondary antibody: HRP-conjugated Goat Anti-Mouse IgG (H + L) (GB23301, Servicebio, 1:2000).

#### 2.10.1 Statistical analysis

All data processing and statistical analyses were performed using R software (Version 4.3.0) and SPSS software (Version 26.0). Normality of continuous variables was assessed using the Shapiro-Wilk test. Continuous variables with a normal distribution were presented as mean ± standard deviation ( $\bar{x}\pm s$), and comparisons between two groups were conducted using the independent samples Student’s t-test. Non-normally distributed continuous data were expressed as median (M) with interquartile range (IQR), and between-group differences were analyzed using the Mann-Whitney U test. Categorical variables were presented as counts (n) and percentages (%), and group comparisons were performed using the chi-square test or Fisher’s exact test, as appropriate. Repeated measures analysis of variance (ANOVA) was used to compare hemodynamic parameters (MAP and HR) at different time points, with post-hoc multiple comparisons adjusted using the Bonferroni correction. All statistical tests were two-sided, and a *P*-value < 0.05 was considered statistically significant.

## 3 Results

### 3.1 Normalization of the GSE179004 dataset

**Figure 1:**
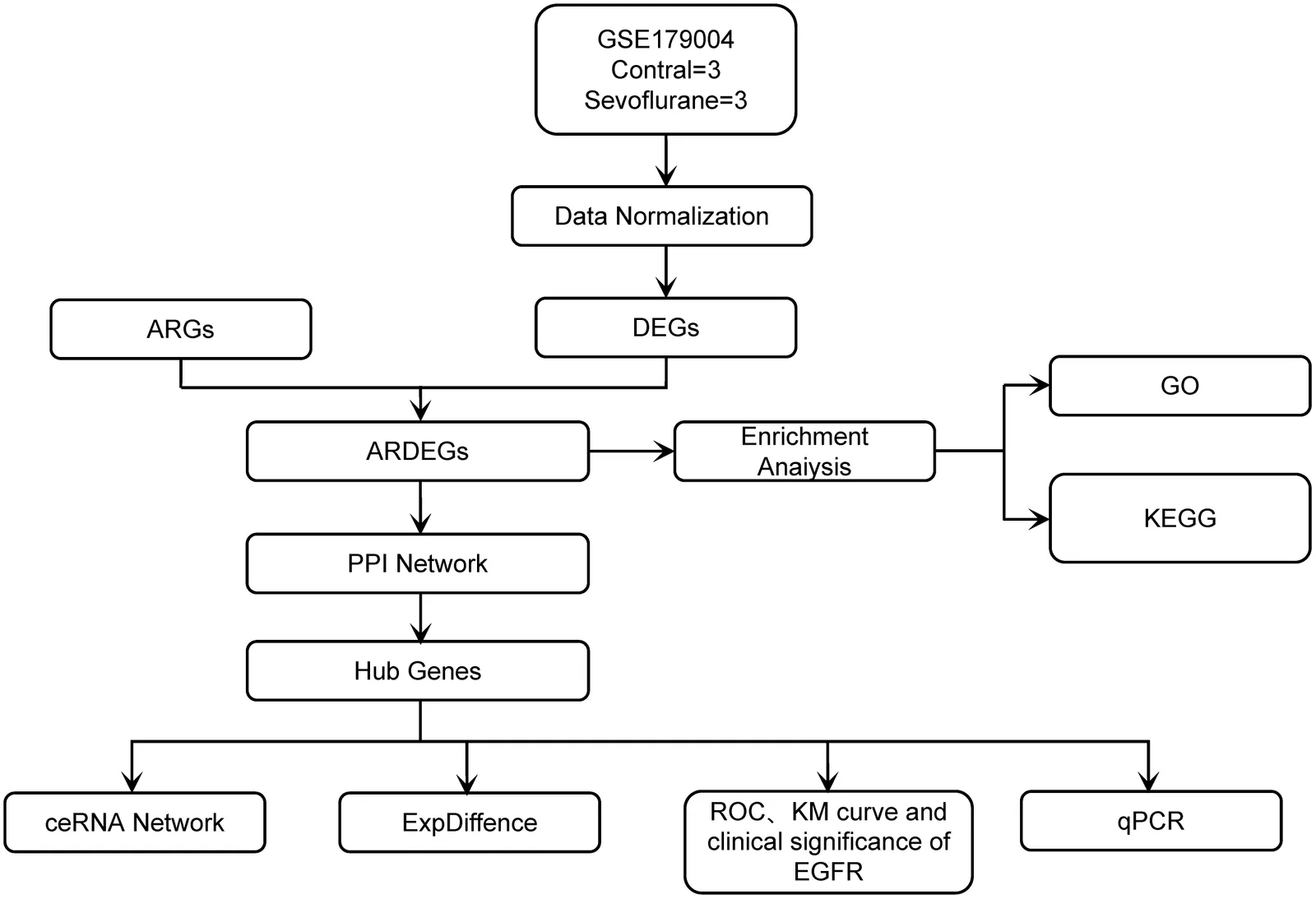
Flowchart of the integrated analysis of ARDEGs.

**Figure 2:**
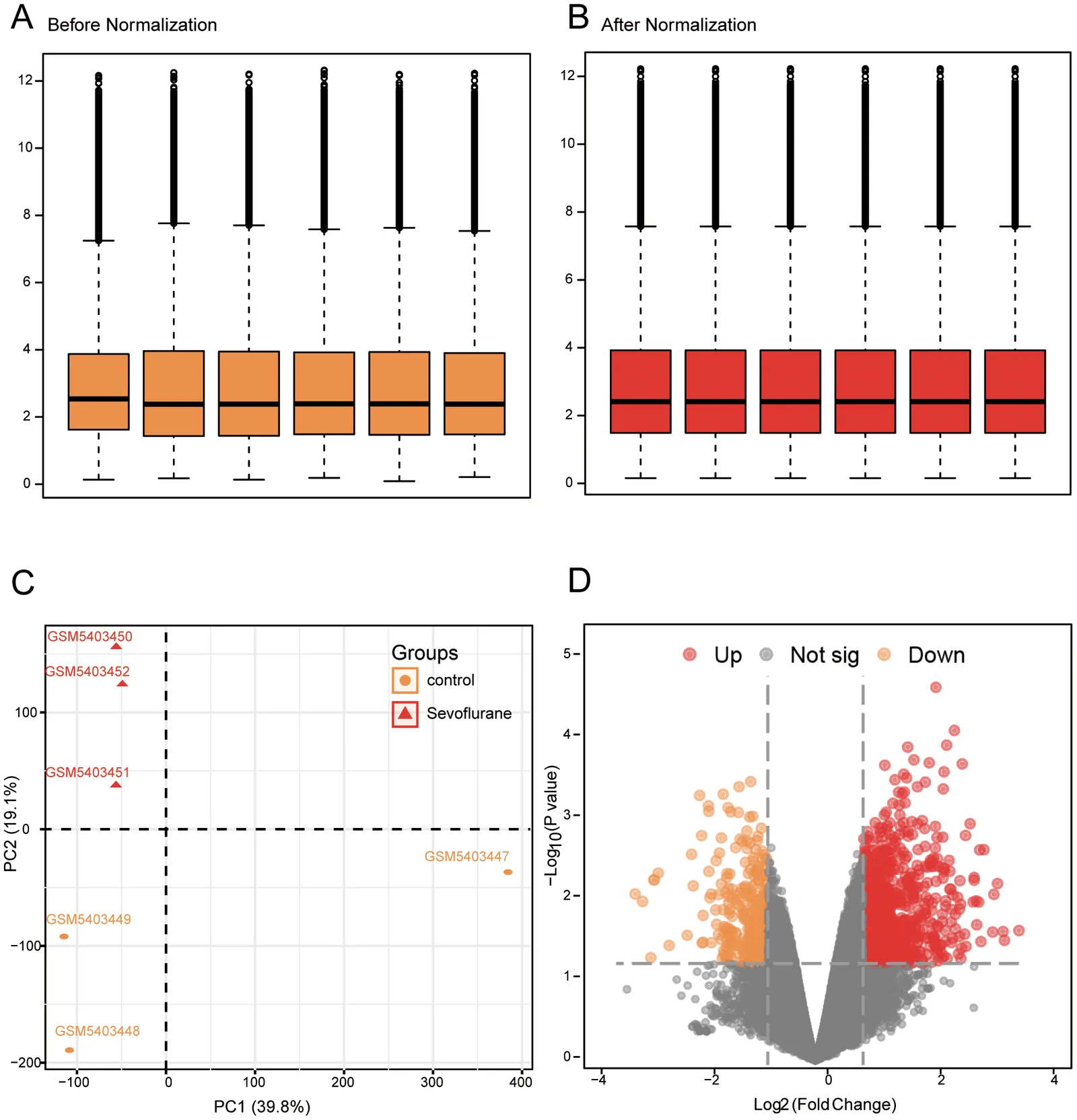
Normalization of the GSE179004 dataset. **(A)** Boxplot of the dataset before normalization. **(B)** Boxplot of the dataset after normalization. **(C)** PCA plot of the dataset. **(D)** Volcano plot of the differential expression analysis.

A flowchart of the integrated bioinformatics and clinical analysis is presented in **Figure 1**. First, the GSE179004 dataset was normalized using the R package sva. The expression values before and after normalization were compared using boxplots (**Figures 2A, B**). Subsequently, PCA was performed, and the low-dimensional distribution of the dataset was visualized in a PCA plot (**Figure 2C**). To identify DEGs between the two groups in the GSE179004 dataset, differential expression analysis was performed using the R package limma. A total of 1,245 genes met the threshold of |logFC| > 1 and *P* < 0.05 (nominal). Among these, 918 genes were upregulated (logFC > 1 and *P* < 0.05) and 327 genes were downregulated (logFC < -1 and *P* < 0.05). It should be noted that these 1,245 genes were identified using a discovery-oriented threshold without genome-wide multiple testing correction at this stage; they were intended to serve as a candidate pool for subsequent functional filtering rather than as a set of robustly validated DEGs. Indeed, after Benjamini-Hochberg correction, the number of genes with adj.*P* < 0.05 was substantially lower, underscoring the exploratory nature of this screening step. The results of the differential expression analysis are presented in a volcano plot (**Figure 2D**).

### 3.2 Identification of angiogenesis-related differentially expressed genes

**Figure 3:**
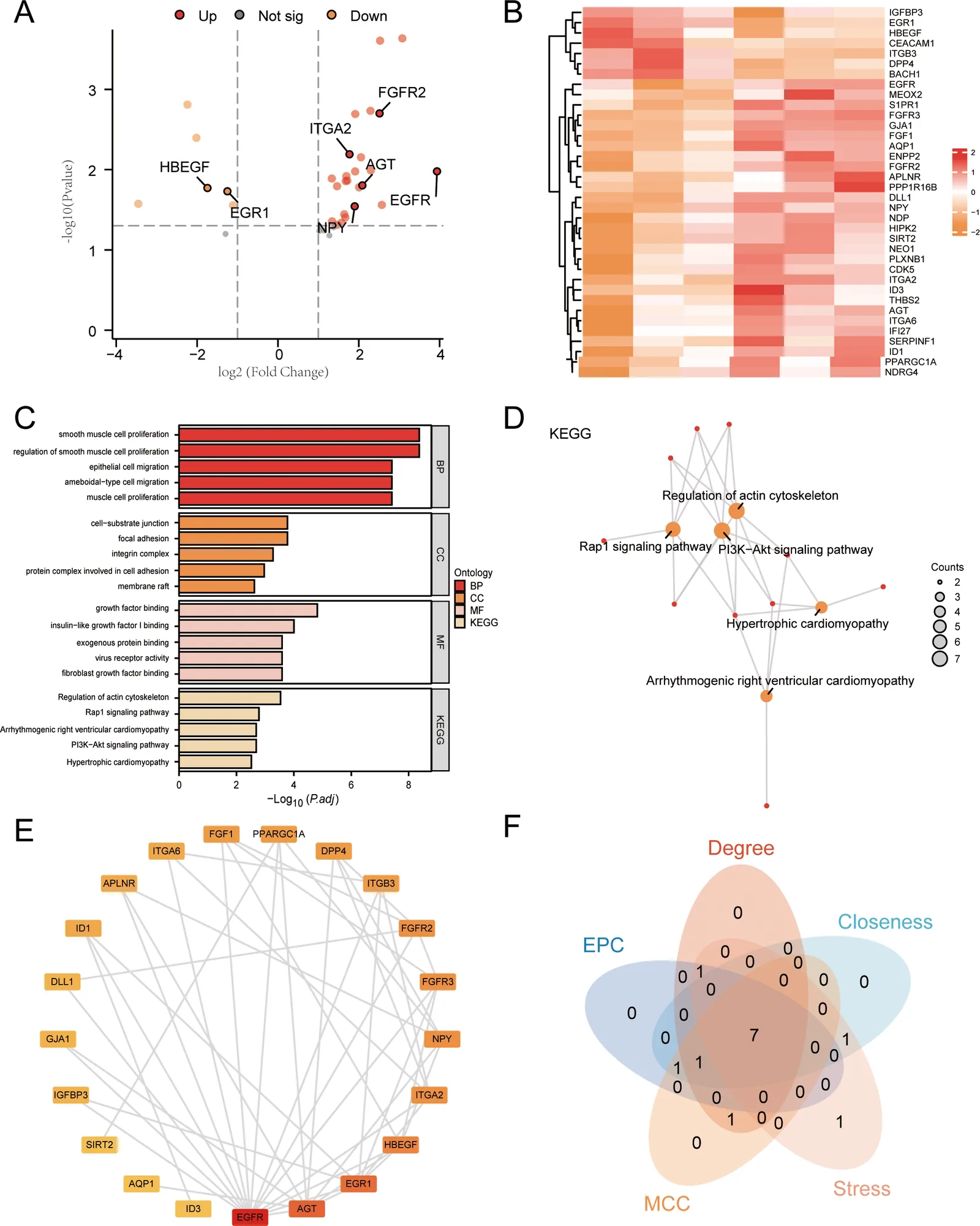
Analysis of angiogenesis-related differentially expressed genes. **(A)** Volcano plot of differentially expressed genes in the ARDEG expression matrix. **(B)** Heatmap showing the expression patterns of the 36 ARDEGs. **(C)** Bar chart illustrating the results of GO and KEGG enrichment analyses for the ARDEGs. **(D)** Network plot of KEGG enrichment analysis for the ARDEGs. Yellow nodes represent pathways, red nodes represent genes, and edges indicate the relationships between pathways and genes. **(E)** PPI network of the ARDEGs constructed using the STRING database. **(F)** Venn diagram of the top 10 ARDEGs identified by each of the five CytoHubba algorithms.

To obtain ARDEGs, an intersection was performed between all DEGs (|logFC| > 1, *P* < 0.05) and the compiled list of ARGs. A total of 36 ARDEGs were identified. Based on these intersection results, the expression levels of the 36 ARDEGs were extracted from the normalized GSE179004 dataset and compiled into a new expression matrix, designated as the ARDEG expression matrix. Using this ARDEG expression matrix, a volcano plot was generated with the hub genes highlighted (**Figure 3A**). Additionally, a heatmap was created using the R package pheatmap to visualize the expression patterns of the ARDEGs (**Figure 3B**).

It is important to note that, following Benjamini-Hochberg correction for multiple testing, none of the 36 ARDEGs retained an adjusted P-value below 0.05. This reflects the limited statistical power inherent to the small sample size of the GSE179004 dataset, which comprised only three samples per group. Accordingly, these 36 genes should be interpreted as candidate ARDEGs identified through a discovery-oriented screening step, rather than as robustly confirmed differential expression signatures. Their biological relevance was subsequently assessed through multi-layered validation, including PPI network analysis, independent TCGA cohort validation, and experimental verification in clinical samples.

To further investigate the functional roles of the 36 ARDEGs, GO and KEGG enrichment analyses were performed. GO enrichment analysis was performed to characterize the functional annotations of the 36 ARDEGs. In the BP category, significantly enriched terms included smooth muscle cell proliferation, regulation of smooth muscle cell proliferation, epithelial cell migration, ameboidal‑type cell migration, muscle cell proliferation, and positive regulation of cell adhesion (adj.*P* < 0.05 for all). In the CC category, the ARDEGs were significantly enriched in cell‑substrate junctions, focal adhesions, integrin complexes, protein complexes involved in cell adhesion, membrane rafts, and cortical actin cytoskeleton (adj.*P* < 0.05 for all). In the MF category, significantly enriched terms included growth factor binding, insulin‑like growth factor binding, exogenous protein binding, virus receptor activity, fibroblast growth factor binding, and heparin binding (adj.*P* < 0.05 for all).

The KEGG pathway enrichment analysis showed that the ARDEGs were significantly enriched in several signaling pathways, including regulation of actin cytoskeleton, the Rap1 signaling pathway, arrhythmogenic right ventricular cardiomyopathy, the PI3K-AKT signaling pathway, and hypertrophic cardiomyopathy. The results of the GO and KEGG enrichment analyses are visualized in a bar chart (**Figure 3C**). Additionally, a KEGG network plot was generated to illustrate the relationships between the enriched pathways and their associated genes (**Figure 3D**). In this network, the connections indicate annotations between genes and pathways, and larger nodes represent pathways enriched with a greater number of genes.

To further explore the interactions among the 36 ARDEGs, PPI network was constructed using the STRING database. The analysis revealed that 21 of the 36 ARDEGs formed an interconnected network, which was subsequently visualized using CytoScape software (**Figure 3E**). To identify key hub genes within this network, five algorithms available in the CytoHubba plugin for CytoScape MCC, Degree, Stress, EPC, and Closeness were applied to score the 21 interconnected ARDEGs. For each algorithm, the genes were ranked based on their scores. The results from the five algorithms were then intersected, and the overlapping genes were visualized using a Venn diagram (**Figure 3F**). These intersecting genes were identified as the angiogenesis-related hub genes. A total of seven hub genes were ultimately identified: *EGFR*, *AGT*, *EGR1*, *HBEGF*, *ITGA2*, *FGFR2*, *NPY*.

### 3.3 Validation of hub gene expression

**Figure 4:**
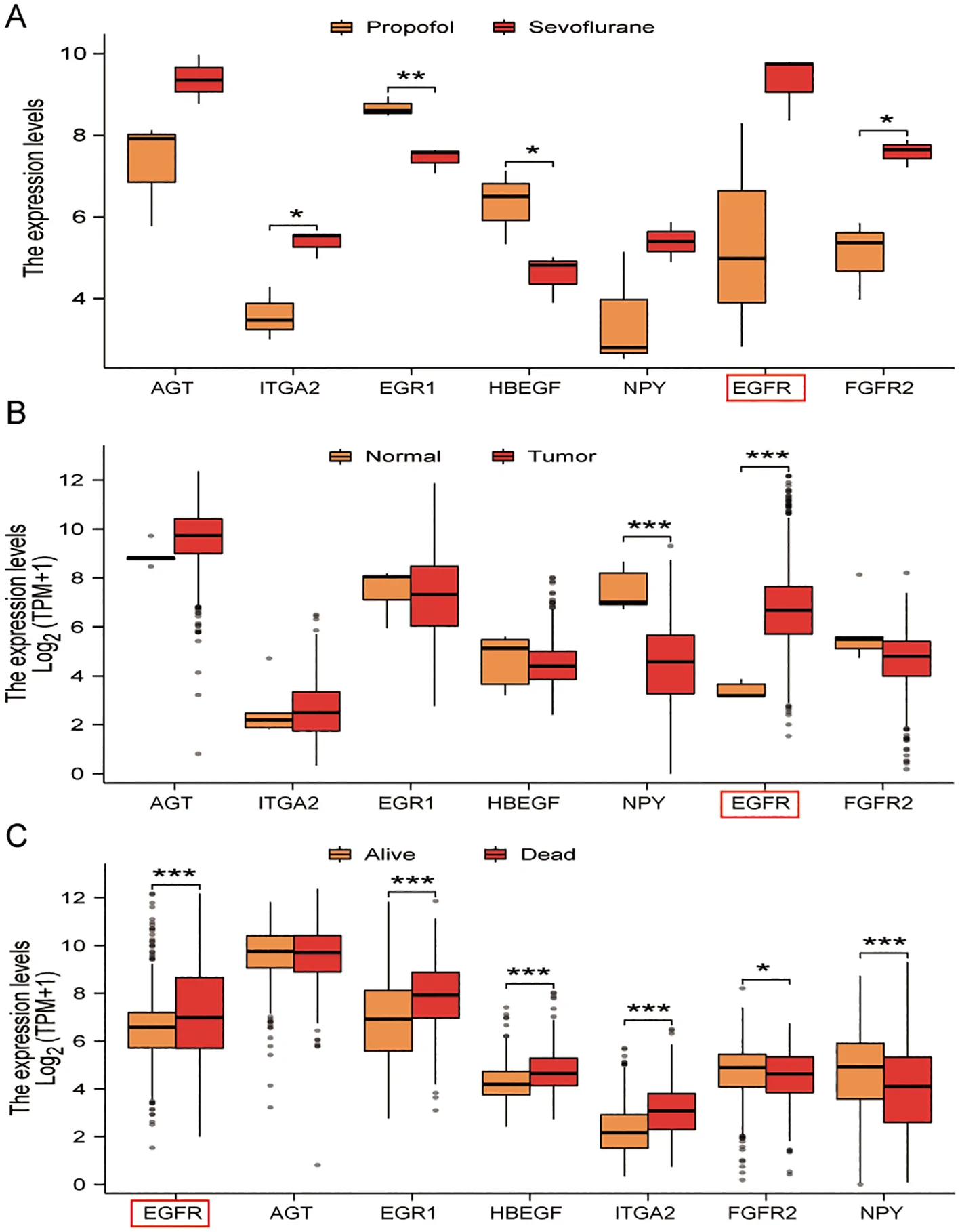
Validation of hub gene expression. **(A)** Comparison of hub gene expression levels between the sevoflurane-treated and control groups in the GSE179004 dataset. **(B)** Comparison of hub gene expression levels between glioma tissues and normal tissues in the TCGA dataset. **(C)** Association between hub gene expression and survival outcomes in glioma patients. *P < 0.05; **P < 0.01; ***P < 0.001.

Differential expression of the seven hub genes was first validated in the GSE179004 dataset, comparing sevoflurane-treated glioma samples with controls (**Figure 4A**). The analysis revealed that *ITGA2* and *FGFR2* were significantly upregulated in the sevoflurane group (*P* < 0.05), while *EGR1* and *HBEGF* were significantly downregulated (*P* < 0.05). No statistically significant differences were observed for *AGT*, *NPY*, or *EGFR* between the two groups (*P* > 0.05).

Comparison between glioma and normal tissues revealed that both NPY and EGFR exhibited statistically significant differential expression. EGFR expression was significantly upregulated in glioma tissues compared to normal controls (*P* < 0.001), whereas NPY expression was significantly downregulated (*P* < 0.001). No significant differences were observed for the remaining hub genes (**Figure 4B**). Furthermore, the association between the expression of these seven hub genes and the survival outcomes of glioma patients was analyzed. The results indicated that *EGFR* expression was significantly elevated in the deceased patient group compared to the surviving group (*P* < 0.001) (**Figure 4C**). This univariable association, however, required further multivariable adjustment to assess whether *EGFR* provides independent prognostic information beyond established molecular classifiers.

### 3.4 EGFR expression and prognosis: univariable and multivariable cox regression analyses

**Figure 5:**
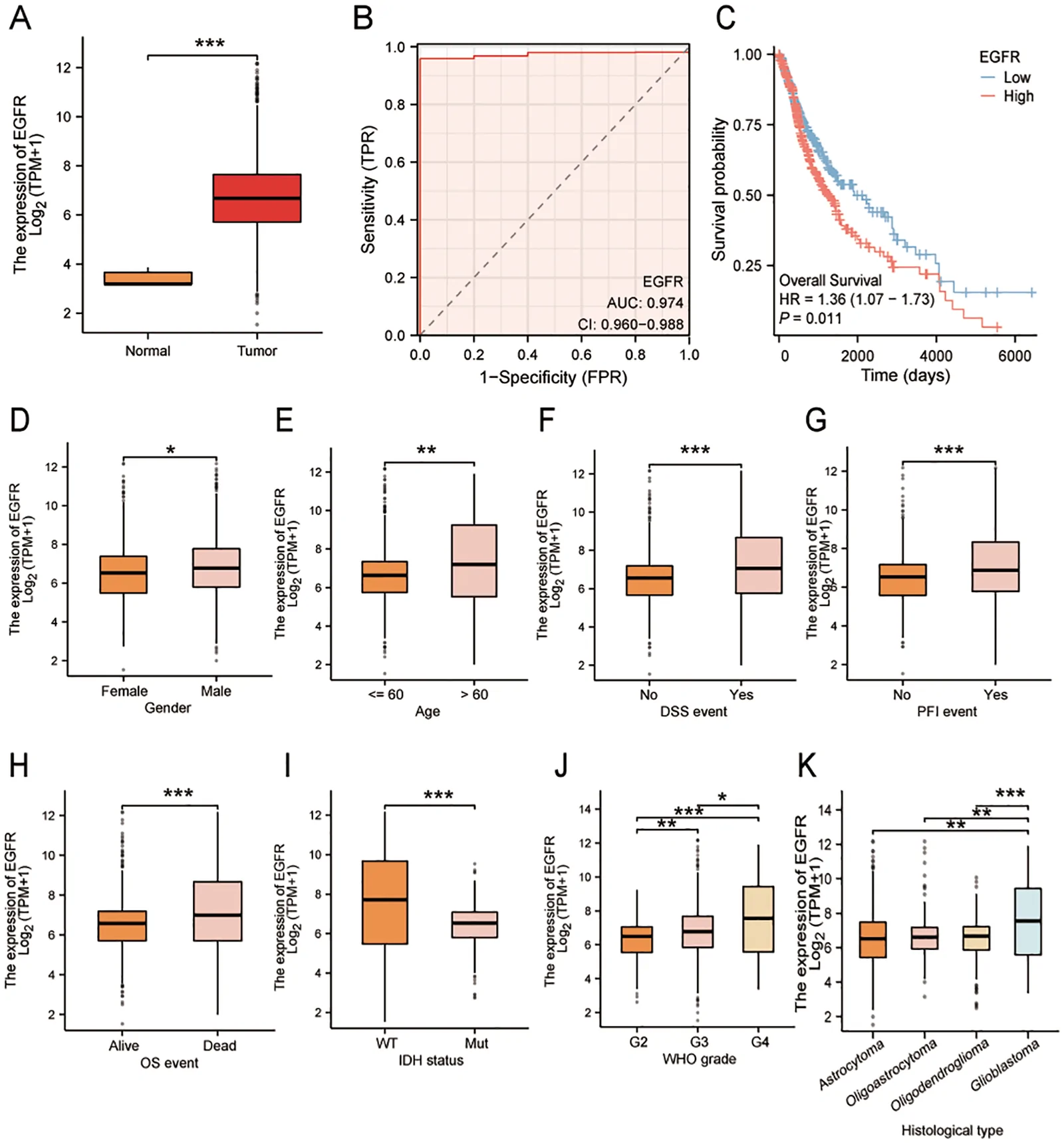
Single-gene analysis of EGFR. **(A)** Differential expression of *EGFR* between glioma tissues and normal tissues. **(B)** ROC curve evaluating the diagnostic value of *EGFR* for glioma prognosis. **(C)** Kaplan-Meier survival curves comparing overall survival between patients with high and low *EGFR* expression. **(D)** *EGFR* expression levels stratified by sex. **(E)** *EGFR* expression levels stratified by age. **(F)** *EGFR* expression levels stratified by disease-specific survival status. **(G)** *EGFR* expression levels stratified by progression-free interval event status. **(H)** *EGFR* expression levels stratified by survival outcome (deceased vs. surviving). **(I)** *EGFR* expression levels stratified by isocitrate dehydrogenase (IDH) mutation status. **(J)** *EGFR* expression levels across different WHO grades. **(K)** *EGFR* expression levels across different glioma subtypes. *P < 0.05; **P < 0.01; ***P < 0.001.

The expression level of *EGFR* was analyzed and compared between glioma tissues and healthy tissues using the TCGA database. The results revealed that *EGFR* expression was significantly elevated in glioma tissues compared to both paired adjacent non-tumor tissues and normal tissues (**Figure 5A**). The performance of EGFR in predicting overall survival status in glioma patients was evaluated using the ROC curve, which yielded an AUC of 0.974, indicating high diagnostic accuracy (**Figure 5B**), indicating excellent prognostic discrimination.

To evaluate the prognostic value of EGFR expression in glioma, we performed univariable and multivariable Cox regression analyses using the combined TCGA-GBM and TCGA-LGG datasets (n = 698). Univariable analysis revealed that high EGFR expression was significantly associated with poor overall survival (HR = 1.364, 95% CI: 1.074-1.734, *P* = 0.011) (**Figure 5C**, **Table 3**). We further examined the relationship between EGFR expression and key clinicopathological features using the TCGA database. High *EGFR* expression was significantly correlated with sex (**Figure 5D**), age (**Figure 5E**), disease-specific survival (**Figure 5F**), progression-free interval events (**Figure 5G**), isocitrate dehydrogenase (IDH) mutation status (**Figure 5I**), and glioma subtype classification (**Figure 5K**) (all *P* < 0.05). Specifically, EGFR expression was significantly higher in deceased patients than in survivors (*P* < 0.001) (**Figure 5H**), and increased progressively with advancing WHO grade from grade II to grade IV (*P* < 0.05) (**Figure 5J**), indicating that *EGFR* expression is elevated in higher-grade, more aggressive gliomas.

**Table 3:** Univariate cox regression.

Characteristics	Total (N)	HR (95% CI)	*P* value
EGFR	698		
Low	349	Reference	
High	349	1.364 (1.074 - 1.734)	0.011
WHO grade	636		
G2	223	Reference	
G3	245	2.967 (1.986 - 4.433)	< 0.001
G4	168	18.600 (12.448 - 27.794)	< 0.001
IDH status	688		
WT	246	Reference	
Mut	442	0.116 (0.089 - 0.151)	< 0.001
1p/19q codeletion	691		
Non-codel	520	Reference	
Codel	171	0.225 (0.147 - 0.346)	< 0.001
Age	698		
<= 60	555	Reference	
> 60	143	4.696 (3.620 - 6.093)	< 0.001

**Figure 6:**
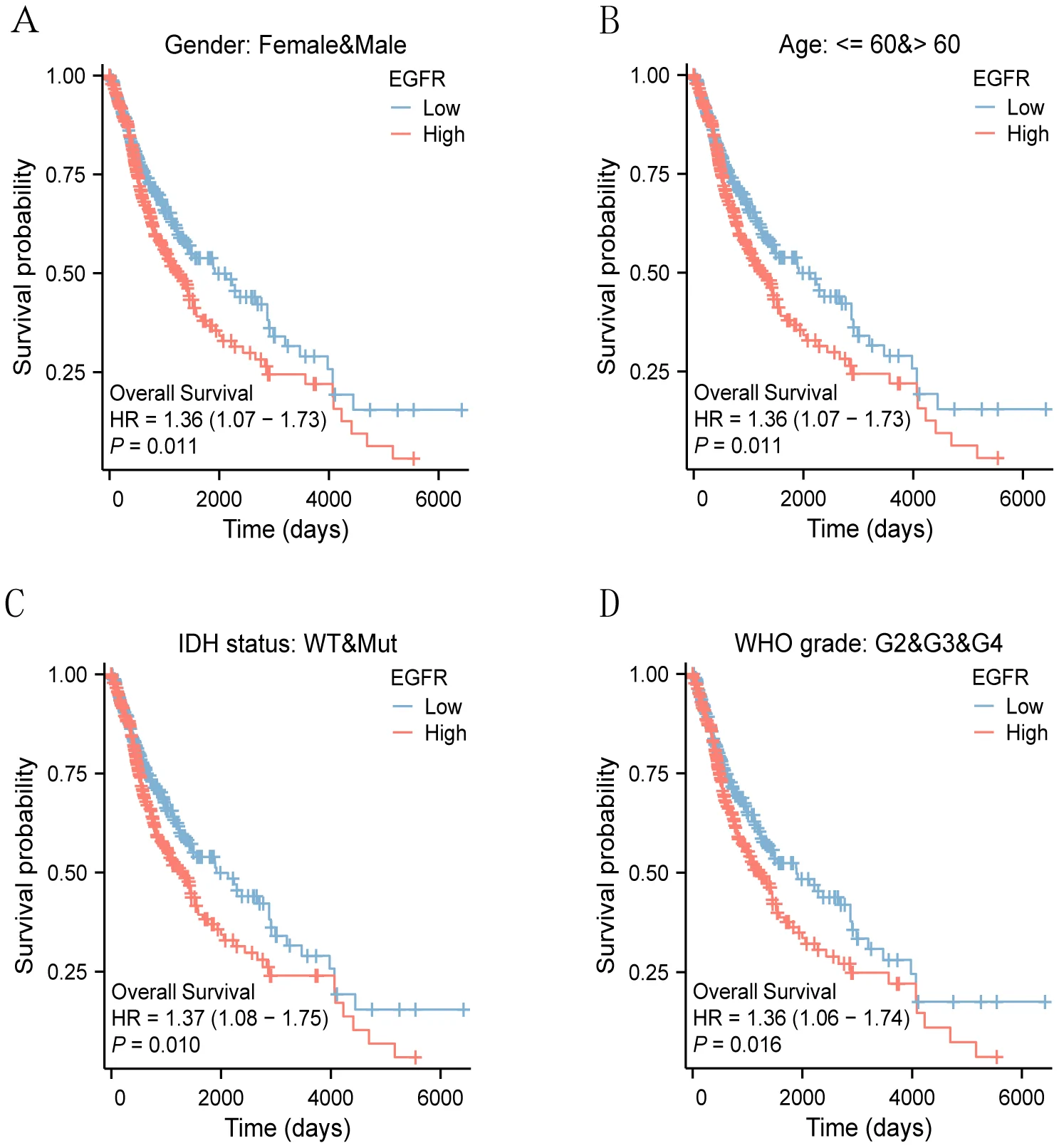
Kaplan–Meier overall survival curves stratified by gender, age, IDH mutation status and WHO pathological grade according to EGFR expression in glioma cohorts. **(A)** Stratification by gender (female and male patients); **(B)** Stratification by age (≤60 years and >60 years); **(C)** Stratification by IDH status (IDH wild-type [WT] and IDH mutant [Mut]); **(D)** Stratification by WHO pathological grade (Grade 2, Grade 3 and Grade 4).

However, after simultaneous adjustment for WHO grade, IDH mutation status, 1p/19q codeletion status, and age in a multivariable Cox model, EGFR high expression was no longer significantly associated with overall survival (HR = 1.008, 95% CI: 0.775-1.313, *P* = 0.950) (**Table 4**). To further evaluate whether the prognostic effect of EGFR differs across subgroups, we performed stratified Kaplan-Meier analyses according to age, sex, IDH status, and WHO grade. Stratified Kaplan-Meier analyses by age, sex, IDH status, and WHO grade revealed consistent HRs across all strata (HR ≈ 1.36-1.37), indicating no significant interaction between EGFR and any single covariate (**Figure 6**).

**Table 4:** Multivariate cox regression.

Characteristics	Total (N)	HR (95% CI)	*P* value
EGFR	698		
Low	349	Reference	
High	349	1.008 (0.775 - 1.313)	0.950
WHO grade	636		
G2	223	Reference	
G3	245	1.963 (1.282 - 3.006)	0.002
G4	168	5.119 (3.069 - 8.540)	< 0.001
IDH status	688		
WT	246	Reference	
Mut	442	0.303 (0.204 - 0.450)	< 0.001
1p/19q codeletion	691		
Non-codel	520	Reference	
Codel	171	0.686 (0.415 - 1.136)	0.143
Age	698		
<= 60	555	Reference	
> 60	143	1.546 (1.132 - 2.112)	0.006

Collectively, these findings suggest that EGFR expression does not provide independent prognostic information beyond established molecular classifiers, particularly IDH status and WHO grade. The apparent survival association observed in univariable analysis most likely reflects its role as a correlate of aggressive molecular subtypes—specifically IDH-wildtype and high-grade gliomas—rather than an independent driver of survival outcomes. Nevertheless, this does not diminish the biological importance of EGFR as a therapeutic target in glioma, particularly in IDH-wildtype glioblastoma, where EGFR-driven signaling pathways retain significant oncogenic activity.

### 3.5 Comparison of baseline characteristics and intraoperative parameters between the two groups

**Figure 7:**
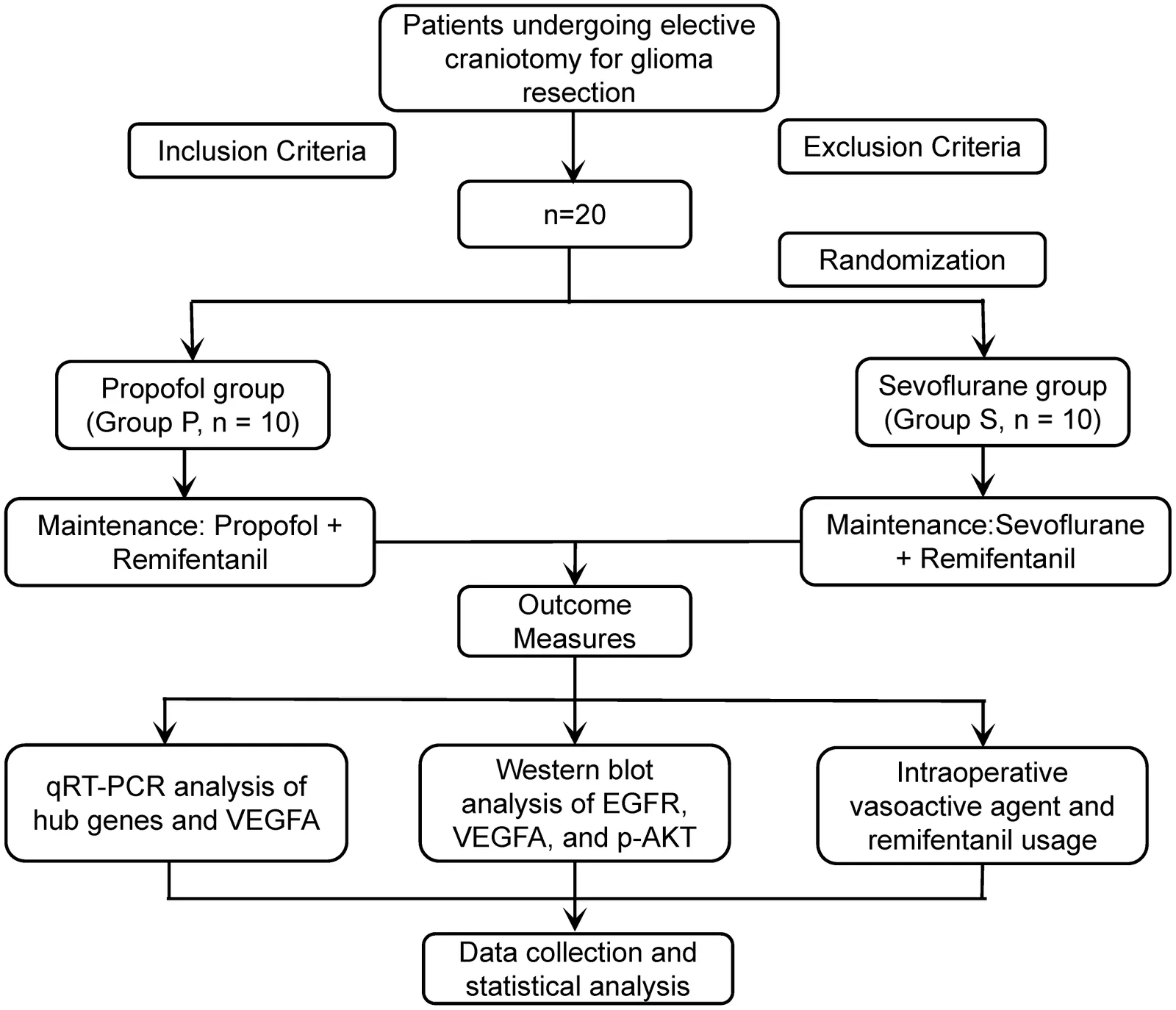
Flowchart of clinical glioma sample collection and analysis.

A total of 20 patients were enrolled in this study according to the predefined selection criteria, with 10 patients assigned to the Group P and 10 to the Group S. No patients withdrew from the study, and data from all 20 participants were included in the final analysis. Postoperative pathological examination confirmed glioblastoma (WHO grade IV) in 9 of 10 patients in the propofol group and 7 of 10 patients in the sevoflurane group. From each group, five glioblastoma cases were randomly selected using a computer-generated random sequence by a researcher not involved in clinical grouping or laboratory testing. The same five specimens per group were used for both qRT-PCR and Western blot analyses (**Figure 7**). There were no statistically significant differences between the two groups in terms of baseline characteristics, including age, sex, BMI, ASA physical status classification, or glioma WHO grade (*P* > 0.05). Regarding intraoperative parameters, no significant differences were observed between the two groups in surgery duration, intraoperative blood loss, urine output, or fluid infusion volume (*P* > 0.05) (**Table 5**).

**Table 5:** Comparison of baseline characteristics and intraoperative parameters between the two groups.

Parameter	Propofol group (n = 10)	Sevoflurane group (n = 10)	*P-*value
Age (years)	52.90 ± 15.70	56.70 ± 14.24	0.285
Sex			1.000
Male	5 (50.0%)	6 (60.0%)	
Female	5 (50.0%)	4 (40.0%)	
BMI (kg/m^2^)	21.40 ± 2.08	22.27 ± 2.02	0.354
ASA Classification			0.170
II	8 (80.0%)	4 (40.0%)	
III	2 (20.0%)	6 (60.0%)	
Glioma WHO grade			0.453
II	0	1	
III	1 (10.0%)	2 (20.0%)	
IV	9 (90.0%)	7 (70.0%)	
Surgery duration (min)	282.00 ± 78.04	354.80 ± 78.56	0.054
Intraoperative blood loss (mL)	275.00 ± 82.50	300.50 ± 78.56	0.477
Intraoperative urine output (mL)	866.00 ± 174.18	975.00 ± 139.94	0.140
Intraoperative fluid infusion (mL)	2340.00 ± 621.74	2890 ± 591.05	0.058

Data for age, BMI, surgery duration, intraoperative blood loss, and intraoperative urine output are presented as $\bar{x}\pm s$. Sex, ASA physical status classification, and glioma WHO grade are presented as number (percentage). A *P*-value < 0.05 was considered statistically significant.

### 3.6 Comparison of intraoperative hemodynamic parameters between the two groups

**Figure 8:**
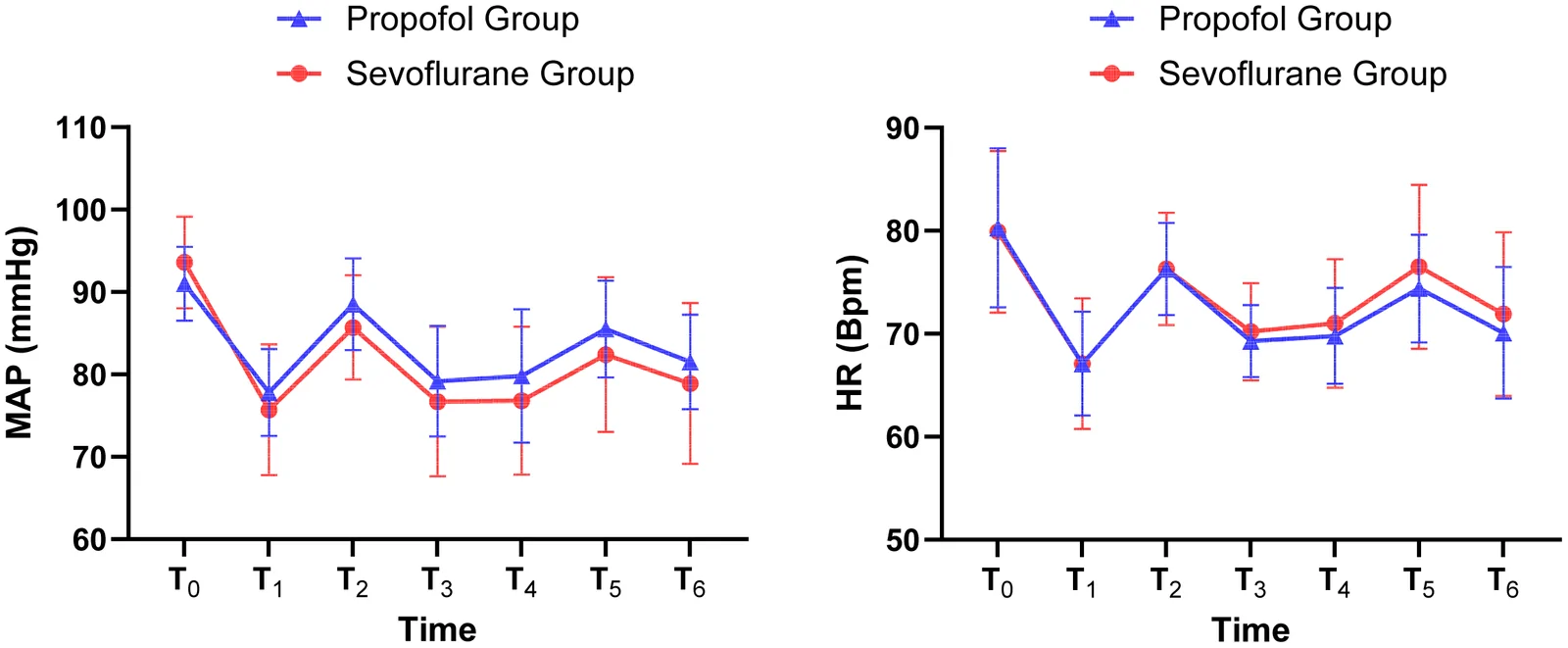
MAP and HR at different time points in the two groups.

Intraoperative MAP and HR at different time points were compared using repeated measures ANOVA. Between-group comparison: no statistically significant differences were observed in MAP or HR between the two groups at any of the measured time points (*P* > 0.05). Within-group comparison: compared to baseline (T_0_), MAP was significantly decreased at T_1_, T_2_, T_3_, T_4_, T_5_, and T_6_ in both groups (*P* < 0.05). Similarly, HR was significantly decreased at T_1_, T_3_, T_4_, and T_6_ compared to T_0_ in both groups (*P* < 0.05) (**Table 6**; **Figure 8**).

**Table 6:** Comparison of MAP and HR between the two groups at different time points.

Time point	Propofol group (n = 10)	Sevoflurane group (n = 10)	F-value	*P*-value
MAP				
T_0_	90.00 ± 4.47	93.60 ± 5.56	F_time_= 49.390 F_group_= 0.451 F_time×group_ = 1.550	*P*_time_ < 0.001 *P*_group_ = 0.510 *P*_time×group_ = 0.169
T_1_	77.80 ± 5.27	75.70 ± 7.89		
T_2_	88.50 ± 5.54	85.70 ± 6.61		
T_3_	79.20 ± 6.71	76.70 ± 9.06		
T_4_	79.80 ± 8.08	76.80 ± 8.97		
T_5_	85.50 ± 5.87	82.40 ± 9.39		
T_6_	81.50 ± 5.72	78.90 ± 9.75		
HR				
T_0_	80.30 ± 7.73	79.90 ± 7.85	F_time_ = 40.527 F_group_ = 0.111 F_time×group_ = 4.650	*P*_time_ < 0.001 *P*_group_ = 0.743 *P*_time×group_ = 0.458
T_1_	67.10 ± 5.04	67.10 ± 6.35		
T_2_	76.30 ± 4.50	76.30 ± 5.46		
T_3_	69.30 ± 3.50	70.20 ± 4.70		
T_4_	69.80 ± 4.64	71.00 ± 6.25		
T_5_	74.40 ± 5.21	76.50 ± 7.93		
T_6_	70.10 ± 6.37	71.90 ± 7.95		

Data are presented as $\bar{x}\pm s$. A *P*-value < 0.05 was considered statistically significant.

### 3.7 Intraoperative use of vasoactive agents and remifentanil

No statistically significant differences were observed between the two groups in the intraoperative use of norepinephrine, nitroglycerin, nimodipine, atropine, or esmolol (*P* > 0.05) (**Table 7**). Similarly, the intraoperative dosage of remifentanil did not differ significantly between the propofol and sevoflurane groups (*P* > 0.05) (**Table 8**).

**Table 7:** Comparison of intraoperative vasoactive agent use between the two groups.

Vasoactive agent	Propofol group (n = 10)	Sevoflurane group (n = 10)	*P*-value
Norepinephrine use	4 (40.0%)	6 (60.0%)	0.656
Nitroglycerin use	1 (10.0%)	0 (0.0%)	1.000
Nimodipine use	0 (0.0%)	0 (0.0%)	1.000
Atropine use	1 (10.0%)	1 (10.0%)	1.000
Esmolol use	0 (0.0%)	0 (0.0%)	1.000

Data are presented as number (percentage). Comparisons were performed using Fisher’s exact test. A *P*-value < 0.05 was considered statistically significant.

**Table 8:** Comparison of remifentanil dosage between the two groups.

Parameter	Propofol group (n = 10)	Sevoflurane group (n = 10)	*P*-value
Remifentanil (g)	1.35 ± 0.32	1.65 ± 0.33	0.056

Data for remifentanil dosage are presented as $\bar{x}\pm s$. A *P*-value < 0.05 was considered statistically significant.

### 3.8 Validation of Hub gene and VEGF-AKT pathway expression by qRT-PCR and Western blot

**Figure 9:**
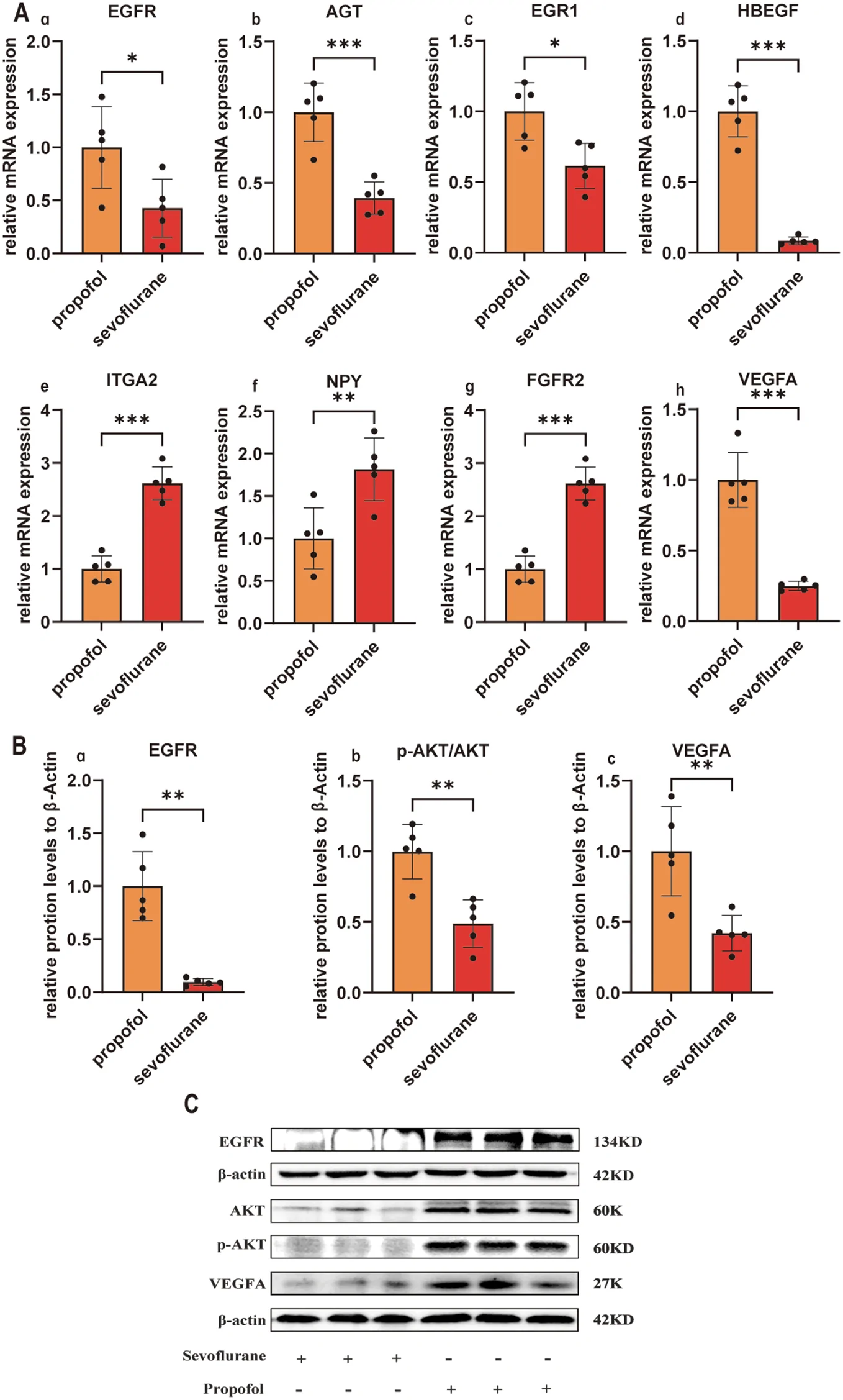
Validation of hub gene and VEGF-AKT pathway expression by qRT-PCR and Western blot. **(A a–h)** qRT-PCR analysis of the expression levels of the seven hub genes (*EGFR*, *AGT*, *EGR1*, *HBEGF*, *ITGA2*, *NPY*, and *FGFR2*) and *VEGFA*. **(B a–d)** Quantitative analysis of Western blot results: **(a)** EGFR protein expression; **(b)** AKT protein expression; **(c)** p-AKT protein expression; **(d)** VEGFA protein expression. **(C)** Representative Western blot images of EGFR, AKT, p-AKT, and VEGFA protein bands. 101, 102, and 103 are the sample numbers for the heptafluorethane group; 001, 002, and 003 are the sample numbers for the isopropylphenol group. Data are presented as $\bar{x}\pm s$. *P < 0.05; **P < 0.01; ***P < 0.001.

qRT-PCR was performed to validate the expression levels of the seven hub genes (*EGFR*, *AGT*, *EGR1*, *HBEGF*, *ITGA2*, *NPY*, and *FGFR2*) in clinical glioma tissue samples. The results demonstrated that, compared to the propofol group, the sevoflurane group exhibited significantly increased expression of *ITGA2* (**Figures 9A-e**), *NPY* (**Figures 9A-f**), and *FGFR2* (**Figures 9A-g**) (*P* < 0.05). In contrast, the expression levels of *EGFR* (**Figures 9A-a**), *AGT* (**Figures 9A-b**), *EGR1* (**Figures 9A-c**), and *HBEGF* (**Figures 9A-d**) were significantly decreased in the sevoflurane group compared to the propofol group (*P* < 0.05). Additionally, the expression of vascular endothelial growth factor A (*VEGFA*) was also found to be significantly downregulated in the sevoflurane group (*P* < 0.05) (**Figures 9A-h**). Western blot analysis was conducted to assess the protein expression levels of EGFR, VEGFA, AKT, and phosphorylated AKT (p-AKT). Consistent with the qRT-PCR results, the protein expression levels of EGFR, VEGFA, and p-AKT/AKT were significantly reduced in the sevoflurane group compared to the propofol group (*P* < 0.05) (**Figure 9B**). Representative blots are shown in **Figure 9C**. After applying the Benjamini-Hochberg false discovery rate (FDR), the 8 mRNA targets and 4 protein targets remained statistically significant (**Table 9**).

**Table 9:** Raw *P*-values and FDR-adjusted q-values for all molecular comparisons

Target	Raw *P*-value	Mean Difference (95% CI)	q-value
EGFR mRNA	0.026323	0.5733 (0.0870, 1.0596)	0.013293
AGT mRNA	0.000434	0.6071 (0.3631, 0.8511)	0.000438
EGR1 mRNA	0.010162	0.3862 (0.1199, 0.6525)	0.005865
HBEGF mRNA	0.000004	0.9151 (0.7271, 1.1031)	0.000007
ITGA2 mRNA	0.000002	-1.987 (-2.3583, -1.6157)	0.000007
NPY mRNA	0.007677	-0.8152 (-1.3470, -0.2834)	0.005865
FGFR2 mRNA	0.000017	-1.617 (-2.0249, -1.2091)	0.000022
VEFGA mRNA	0.010162	0.3862 (0.1199, 0.6525)	0.005865
EGFR protein	0.000268	0.9044 (0.5663, 1.2425)	0.001081
VEGFA protein	0.002205	0.5793 (0.2290, 0.9296)	0.002969
AKT protein	0.002205	0.5652 (0.2707, 0.8597)	0.001822
p-AKT protein	0.000902	0.7840 (0.4312, 1.1368)	0.005180

Multiple comparisons were corrected using the Benjamini-Hochberg false discovery rate (FDR) method across 12 comparisons. A q-value < 0.05 was considered statistically significant.

## 4 Discussion

As an exploratory pilot study, our primary objective was to generate hypotheses regarding the association between sevoflurane and molecular changes in glioma tissues, aimed to investigate whether sevoflurane is associated with expression changes in EGFR-VEGF-AKT pathway components in human glioma tissues. Our results demonstrated that sevoflurane was associated with significantly reduced expression levels of EGFR, VEGFA, and p-AKT, suggesting potential modulation of the EGFR-VEGF-AKT signaling axis. Furthermore, no significant differences in intraoperative hemodynamic parameters were observed between patients receiving sevoflurane anesthesia and those receiving propofol, indicating that sevoflurane has a favorable safety and tolerability profile in this clinical setting.

Gliomas are common and highly aggressive primary malignant tumors with limited treatment options and a poor prognosis (37). The PI3K/AKT signaling pathway is one of the most frequently hyperactivated pathways in glioma, driving not only tumor cell proliferation and survival but also angiogenesis through the upregulation of VEGFA and other pro-angiogenic factors (38). Accurately assessing the impact of anesthetic agents on the tumor microenvironment is therefore of great significance for optimizing perioperative management in glioma patients. However, previous studies have largely focused on individual molecules such as VEGF or HIF-1α, with limited investigation into upstream regulators and broader signaling pathways (39). In the present study, by integrating differential expression analysis of the GEO dataset, angiogenesis-related genes, and PPI network analysis, we identified seven key genes, including *EGFR*, *AGT*, and *EGR1*, and propose that *EGFR* may serve as an upstream regulatory target mediating the anti-angiogenic effects of sevoflurane. Unlike bevacizumab, which exerts its effects by directly targeting VEGF (40–42), sevoflurane appears to simultaneously modulate EGFR, VEGF, and downstream AKT signaling molecules. This multi-target, pathway-level inhibitory effect may confer a broader therapeutic potential for sevoflurane in anti-angiogenic treatment strategies for glioma.

To further explore the potential molecular mechanisms underlying the effect of sevoflurane on glioma‑associated angiogenesis, we performed bioinformatics analysis on the GSE179004 dataset. Through differential expression analysis, a total of 1,245 DEGs associated with sevoflurane treatment were identified. By intersecting these DEGs with angiogenesis‑related genes, we successfully obtained 36 candidate ARDEGs. GO and KEGG enrichment analyses were conducted on the ARDEGs to investigate the molecular mechanisms by which sevoflurane affects glioma‑associated angiogenesis. GO enrichment analysis revealed that these genes were primarily enriched in biological processes such as “regulation of smooth muscle cell proliferation”. Previous studies have shown that the maturation and stability of blood vessel walls depend on the coverage of vascular smooth muscle cells and pericytes. The proliferation and migration of smooth muscle cells are critical steps in neovascularization and functionalization, and smooth muscle cell proliferation is essential for vascular development and stability (43). Sevoflurane may interfere with these processes, thereby inhibiting the formation of a functional vascular network by compromising the structural integrity and functional stability of newly formed blood vessels. KEGG enrichment analysis further identified the PI3K-AKT signaling pathway as one of the key pathways involved in the action of sevoflurane. Aberrant activation of this pathway is closely associated with glioma progression and therapeutic resistance (44). Meanwhile, VEGF, a core regulator of angiogenesis, promotes endothelial cell proliferation and migration by activating VEGF receptors and downstream signaling pathways (43). These bioinformatics predictions strongly suggest that sevoflurane may exert its anti-angiogenic effects by interfering with key signaling networks centered on the PI3K-AKT pathway that are involved in vascular development and homeostasis.

Subsequent validation using human glioma tissue specimens provided strong experimental support for the bioinformatics predictions. The experimental results showed that, compared with the propofol group, sevoflurane treatment significantly reduced the protein levels of VEGF and p-AKT in glioma tissues. This finding directly confirms the inhibitory effect of sevoflurane on the VEGF-AKT signaling axis, which is highly consistent with the hypothesis proposed by the bioinformatics analysis that sevoflurane may affect downstream angiogenic processes by inhibiting the VEGF-AKT pathway. This observation is strongly supported by multiple independent studies. Gao et al. demonstrated that sevoflurane inhibits proliferation, invasion, and migration while promoting apoptosis in glioma cells through downregulation of the PI3K/AKT signaling pathway (45).Wang et al. showed that sevoflurane inhibits VEGF and bFGF stimulated endothelial cell migration, adhesion, and growth through suppression of Ras/Akt/mTOR signaling (46).Yi et al. further revealed that sevoflurane upregulates miRNA-637, which suppresses Akt1 expression and activity, thereby inhibiting glioma cell migration and invasion (47).Collectively, these findings establish PI3K/AKT as a converging node through which sevoflurane exerts its pleiotropic effects on glioma cells and the tumor microenvironment. This is also consistent with results from studies on breast and lung cancer, in which downregulation of the VEGF and HIF-1α signaling pathways was shown to inhibit tumor angiogenesis and subsequently delay tumor progression (22, 48).

Through PPI network analysis and hub gene screening, we identified seven key genes, including *EGFR*, *AGT*, and *EGR1*, with *EGFR* emerging as a prioritized candidate potentially involved in the observed molecular changes associated with sevoflurane exposure. The changes in EGFR expression were subsequently validated in clinical specimens. EGFR is a tyrosine kinase receptor that plays a critical role in regulating cell growth, survival, proliferation, and differentiation. In our study, EGFR was identified as one of the key genes significantly associated with glioma angiogenesis. Analysis of the TCGA database confirmed that EGFR expression levels were significantly higher in glioma tissues than in normal brain tissues, and that high EGFR expression was closely associated with higher WHO grade and IDH wild-type status. However, our multivariable Cox regression analysis revealed that the prognostic significance of EGFR observed in univariable analysis was substantially attenuated after simultaneous adjustment for WHO grade, IDH status, 1p/19q codeletion, and age. Stratified analyses further demonstrated that EGFR’s association with survival was consistent across age, sex, IDH, and WHO subgroups, indicating the absence of significant interaction with any single covariate. Collectively, these findings suggest that EGFR expression level does not provide independent prognostic information beyond established molecular classifiers. The apparent survival association of *EGFR* in univariable analysis most likely reflects its role as a correlate of aggressive molecular subtypes (particularly IDH-wild-type and high-grade gliomas) rather than an independent driver of survival outcomes. The ROC curve analysis indicated good discriminative ability for 1-year survival status classification, but this should not be interpreted as evidence for independent prognostic value. Nevertheless, these findings do not diminish the biological importance of EGFR as a therapeutic target in glioma, particularly in the IDH-wild-type GBM subset where EGFR-driven signaling pathways remain actively oncogenic. This finding is consistent with previous studies demonstrating that EGFR overexpression promotes tumorigenesis and therapeutic resistance in various cancers, including glioma (49, 50). Notably, after sevoflurane treatment in glioma patients, the change in *EGFR* expression did not reach statistical significance, contrasting with the trend of high *EGFR* expression originally observed in glioma samples, suggesting that sevoflurane inhibits *EGFR* expression to a certain extent. To ensure the reliability of the laboratory test results, we randomly selected five cases of postoperatively confirmed glioblastoma tissue samples from each of the two groups for qRT-PCR and Western blot analysis, thereby conducting a preliminary investigation into the potential mechanisms by which sevoflurane anesthesia affects molecular markers related to glioma angiogenesis. In the validation experiment using human glioma tissue specimens, we found that the levels of EGFR and VEGF mRNA as well as p-AKT protein expression were significantly lower in the sevoflurane group than in the propofol group, suggesting that sevoflurane may inhibit angiogenesis through suppression of the EGFR-VEGF-AKT signaling pathway. The centrality of the PI3K/AKT pathway in glioma angiogenesis is further underscored by recent studies demonstrating that lncRNA WARS2-IT1 promotes glioma angiogenesis through activation of PI3K/AKT signaling via the miR-299-3p/VEGFA axis (51), and that targeting PI3K/AKT effectively disrupts pro-angiogenic signaling in the glioma microenvironment (38).This finding aligns with current research directions in anti-tumor therapy targeting the EGFR signaling pathway (52, 53), generating hypotheses regarding the potential application of sevoflurane in glioma therapy.

Notably, differences in EGFR expression trends were observed between the GEO dataset (GSE179004) and clinical samples. GEO data showed no significant change in EGFR expression after sevoflurane treatment, whereas clinical samples exhibited downregulation. This discrepancy may be due to several factors. First, sample size: the GEO dataset included only three treated and three control samples, limiting statistical power. In contrast, clinical validation used strict randomization and blinding, enhancing reliability despite only five cases per group. Second, sample heterogeneity: differences in tumor grade, IDH mutation status, and baseline characteristics between GEO and clinical samples may affect *EGFR* expression and its response to sevoflurane. Third, detection methods: GEO used microarrays, while clinical samples were validated with qRT-PCR and Western blot, which offer greater sensitivity and specificity. Overall, the clinical validation more accurately reflects the regulatory effect of sevoflurane on *EGFR*, consistent with previous reports in other tumor types.

It should be acknowledged that propofol, used as the comparator anesthetic in this study, is not a biologically inert control. Accumulating evidence indicates that propofol exerts anti-tumor and anti-angiogenic effects in various malignancies, including glioma, through modulation of multiple signaling pathways (54–56). Notably, sevoflurane still demonstrated stronger suppression of EGFR-VEGF-AKT signaling compared to propofol, even though propofol is already known to possess potential inhibitory activity. This observation may actually reinforce the biological relevance of our findings. Nevertheless, the absence of a true untreated control group limits our ability to delineate the absolute effects of sevoflurane relative to propofol, and future studies incorporating such a control arm are needed.

The differential concordance patterns across the seven hub genes warrant further discussion. The four genes showing consistent GEO-to-clinical directionality (*ITGA2, FGFR2, EGR1, HBEGF*) represent the most robust candidates for sevoflurane-associated expression changes that are reproducible across different study settings. ITGA_2_ and FGFR2 are both involved in cell adhesion and growth factor signaling, and their consistent upregulation with sevoflurane may reflect a shift toward a less proliferative or invasive phenotype. EGR1 and HBEGF are both immediate-early response genes involved in growth factor signaling; their consistent downregulation with sevoflurane aligns with the overall suppression of EGFR-VEGF-AKT signaling. The discordant genes (*EGFR, AGT, NPY*) highlight important limitations of our approach. EGFR’s emergence as a hub gene was driven more by network centrality and biological plausibility than by differential expression in the GEO discovery dataset. NPY upregulated by sevoflurane in clinical tissue but downregulated in glioma versus normal brain in TCGA, this suggests a complex role that may involve context-dependent effects on angiogenesis, neuropeptide signaling, or both. These discordant findings underscore the need for mechanistic studies to delineate the functional consequences of sevoflurane-induced gene expression changes and to determine whether they translate into meaningful biological effects on glioma angiogenesis and progression.

Sevoflurane is an inhalational anesthetic that has been used in clinical practice for over 30 years, with well-characterized pharmacokinetic properties, adverse effect profile, and established safety dosage range (57). On the basis of preliminarily validating the expression changes of bioinformatically screened key genes in human glioma samples, this study further investigated the cerebrovascular safety of sevoflurane during glioma surgery and evaluated its feasibility as a perioperative anesthetic. Existing evidence indicates that the effects of sevoflurane on cerebral hemodynamics are dose-dependent. At 0.5 MAC (0.8-1%) and 1.5 MAC (2.2-3%), sevoflurane dilates cerebral blood vessels, increases blood flow velocity in the middle cerebral artery, and elevates intracranial pressure while reducing cerebrovascular resistance (58). Due to the potential risk of increased intracranial pressure associated with inhaled anesthetics, their use in neurosurgical anesthesia was once controversial. However, recent studies have shown that sevoflurane offers distinct advantages over total intravenous anesthesia in maintaining systemic hemodynamic stability, promoting anesthetic recovery, and preserving cerebral autoregulation (59). Continuous administration of 1.5% sevoflurane for 60 minutes, or 3% sevoflurane for 30 to 60 minutes, significantly improves neurobehavioral function, alleviates cerebral edema, and reduces neuronal death in the basal cortex (60, 61).

This study provides direct clinical evidence for the safety of sevoflurane in neurosurgical procedures. Regarding anesthesia management, both groups received a BIS-guided precision anesthesia strategy, ensuring uniformity of anesthetic depth. Intraoperative hemodynamic monitoring data showed that although the intensity of surgical stimuli varied across different stages (e.g., skin incision, dural opening, tumor resection), there were no significant differences in MAP or HR between the sevoflurane group and the propofol group at any time point from T_0_ to T_6_. Furthermore, no statistically significant differences were observed between the two groups in the rate of vasoactive drug use or remifentanil consumption. These findings indicate that at clinically reasonable concentrations, no significant differences in MAP or HR were observed between the sevoflurane and propofol groups at any measured time point; its cerebrovascular effects remain within a controllable range and do not adversely affect systemic hemodynamics, which is consistent with previous results (62). Moreover, we can infer that the differences in molecular expression between the two groups are primarily attributable to the distinct pharmacological properties of sevoflurane and propofol, rather than to interferences from perioperative hemodynamic status, stress levels, or analgesic intensity.

However, several limitations of this study should be acknowledged. First, the sample size is small. The bioinformatic discovery phase was based on a single GEO dataset (GSE179004), comprising only three sevoflurane-treated and three control samples. This extremely limited sample size substantially reduces statistical power and elevates the risk of false-positive findings, particularly in the context of genome-wide differential expression analyses. The current scarcity of additional public datasets that simultaneously meet both the “sevoflurane treatment” and “glioma” criteria represents an inherent constraint of this study. Future investigations incorporating larger sample sizes and multi-center transcriptomic cohorts are urgently needed to substantiate and refine our preliminary findings. And the clinical validation included only 20 patients (10 per group). This may compromise statistical power and limit subgroup analyses, posing a risk of selection bias. ASecond, there is a lack of cellular and animal experimental validation. Although bioinformatics analysis and clinical samples confirmed the relevant molecular changes, the anti-angiogenic effect of sevoflurane and its pathway specificity have not been directly verified through *in vitro* endothelial cell proliferation and migration assays or *in vivo* glioma xenograft models. Third, long-term clinical follow-up is absent. This study only detected molecular expression changes in intraoperative tumor tissues and did not track key clinical outcomes such as postoperative recurrence rate, progression-free survival, or overall survival. Therefore, the impact of sevoflurane on long-term patient prognosis remains unclear. Fourth, we did not perform systematic molecular subtyping, including assessment of IDH mutation status, MGMT promoter methylation, and EGFR amplification, nor did we conduct quantitative histopathological evaluation of key features such as tumor necrosis and microvessel density in the selected samples. These covariates are well-established confounders of EGFR expression and patient prognosis in glioma, and their absence limits our ability to assess whether the effects of sevoflurane differ across molecular subtypes. Future prospective studies with comprehensive molecular profiling are needed to address this question. Fifth, and most critically, this study lacks direct functional assays for angiogenesis. The downregulation of EGFR, VEGFA, AKT, and p-AKT was measured in bulk glioma tissues, which precludes direct conclusions about endothelial cell proliferation, migration, tube formation, or microvessel density. Thus, our molecular-level findings provide associative evidence but do not constitute functional proof of angiogenesis inhibition. Future studies incorporating CD31/CD34-based microvessel density quantification, endothelial cell functional assays, and *in vivo* glioma xenograft models are essential to determine whether these molecular changes translate into genuine anti-angiogenic effects. Furthermore, differences in the composition of immune cells and stromal cells within the tumor microenvironment may also affect the overall response of tumors to anesthetics. This study did not conduct in-depth analyses at the single-cell or spatial transcriptomic level to address these issues.

Additionally, a critical question remains whether short-term exposure to sevoflurane during surgery (typically lasting several hours) can produce sustained molecular changes that meaningfully affect long-term glioma prognosis. Although we observed downregulation of EGFR, VEGF, and p-AKT in tumor tissues collected at the end of surgery, the persistence of these changes and their biological significance in residual tumor cells remain unknown. Future animal studies with extended postoperative observation periods are needed to address this question.

Despite these limitations, this pilot study provides preliminary associative evidence that sevoflurane is associated with reduced EGFR-VEGF-AKT signaling in human glioma tissues, generating hypotheses for future mechanistic investigations into its potential anti-angiogenic effects. It also verifies the safety and preliminary efficacy of sevoflurane in clinical samples, thereby providing an important theoretical basis for subsequent investigations. In the future, multicenter, large-sample, prospective randomized controlled trials with extended follow-up periods should be conducted to clarify the impact of sevoflurane anesthesia on the long-term prognosis of patients with different glioma subtypes. Furthermore, by establishing various glioma cell lines and animal models combined with conditional gene knockout technology, the mechanism by which sevoflurane inhibits angiogenesis through regulation of the EGFR-VEGF-AKT axis should be thoroughly elucidated at the cellular and *in vivo* levels. The synergistic effects of sevoflurane with existing therapies, as well as its personalized application pathways, should also be explored to validate the findings of this study.

## 5 Conclusion

In this exploratory pilot molecular association study comparing sevoflurane with propofol, propofol is a comparator anesthetic with established anti-tumor properties. Sevoflurane was associated with relatively lower expression of EGFR, VEGFA, and p-AKT in human glioma tissues, suggesting differential modulation of the EGFR-VEGF-AKT signaling axis. Owing to the limited sample size, absence of an anesthesia-free control arm, lack of long-term follow-up, and absence of functional angiogenesis assays, these findings remain hypothesis-generating and require validation in larger cohorts with appropriate control groups and comprehensive molecular stratification.

## Data Availability

The original contributions presented in the study are included in the article/**Supplementary Material**. Further inquiries can be directed to the corresponding authors.
